# *In Vivo* Binding and Retention of CD4-Specific DARPin 57.2 in Macaques

**DOI:** 10.1371/journal.pone.0012455

**Published:** 2010-08-27

**Authors:** Pavel Pugach, Anders Krarup, Agegnehu Gettie, Marcelo Kuroda, James Blanchard, Michael Piatak, Jeffrey D. Lifson, Alexandra Trkola, Melissa Robbiani

**Affiliations:** 1 Center for Biomedical Research, Population Council, New York, New York, United States of America; 2 Division of Infectious Diseases, University Hospital Zurich, Zurich, Switzerland; 3 Aaron Diamond AIDS Research Center, The Rockefeller University, New York, New York, United States of America; 4 Tulane National Primate Research Center, Tulane University Health Sciences Center, Covington, Louisiana, United States of America; 5 AIDS and Cancer Virus Program, SAIC-Frederick, Inc., National Cancer Institute at Frederick, Maryland, United States of America; New York University, United States of America

## Abstract

**Background:**

The recently described Designed Ankyrin Repeat Protein (DARPin) technology can produce highly selective ligands to a variety of biological targets at a low production cost.

**Methodology/Principal Findings:**

To investigate the *in vivo* use of DARPins for future application to novel anti-HIV strategies, we identified potent CD4-specific DARPins that recognize rhesus CD4 and followed the fate of intravenously injected CD4-specific DARPin 57.2 in rhesus macaques. The human CD4-specific DARPin 57.2 bound macaque CD4^+^ cells and exhibited potent inhibitory activity against SIV infection *in vitro*. DARPin 57.2 or the control E3_5 DARPin was injected into rhesus macaques and the fate of cell-free and cell-bound CD4-specific DARPin was evaluated. DARPin-bound CD4^+^ cells were detected in the peripheral blood as early as 30 minutes after the injection, decreasing within 6 hours and being almost undetectable within 24 hours. The amount of DARPin bound was dependent on the amount of DARPin injected. CD4-specific DARPin was also detected on CD4^+^ cells in the lymph nodes within 30 minutes, which persisted with similar kinetics to blood. More extensive analysis using blood revealed that DARPin 57.2 bound to all CD4^+^ cell types (T cells, monocytes, dendritic cells) *in vivo* and *in vitro* with the amount of binding directly proportional to the amount of CD4 on the cell surface. Cell-free DARPins were also detected in the plasma, but were rapidly cleared from circulation.

**Conclusions/Significance:**

We demonstrated that the CD4-specific DARPin can rapidly and selectively bind its target cells *in vivo*, warranting further studies on possible clinical use of the DARPin technology.

## Introduction

Antibodies, generated *in vivo* or by selection from libraries *in vitro*, are widely used for the treatment of a variety of conditions, such as malignancies, transplant rejection and autoimmune disorders [1], [2]. However, antibodies have relatively high production costs and depend on intact disulfide bonds for stability. In contrast, recently described Designed Ankyrin Repeat Proteins (DARPins) are immunologically silent, have superior stability and can be cheaply produced in prokaryotic systems, while maintaining high selectivity and affinity for the target that they are selected against [3], [4]. DARPin technology is already effectively used in biomedical research and potentially could be used in treatment and prevention of disease, including HIV infection.

Ankyrin repeat proteins are a family of proteins that are found across multiple species and mediate protein-protein interactions in various cell compartments [5]. They are composed of stacked, 33 amino acid repeats, each forming a β-turn that is followed by two antiparallel α-helices and a loop reaching the β-turn of the next repeat [6]. These repeats are flanked by constant capping regions, forming one contiguous polypeptide chain. Target protein binding involves contact through the tips of the β-hairpins and the surface of the helical bundle facing the concave ankyrin groove [7]. A library of DARPins was created by varying the number of repeats and randomizing the seven non-structural residues within the repeats [4]. We have previously characterized a panel of CD4-specific DARPins, which efficiently inhibit infection by a broad range of HIV strains and provided preliminary evidence that human CD4-specific DARPins bound macaque CD4 and inhibited SIV replication *in vitro*, without affecting the CD4^+^ memory T cell function [8].

Determining the fate of the DARPins in a relevant animal model is critical for the advancement of this promising technology for future topical clinical applications against HIV. Here, for the first time, we describe the fate of the DARPins *in vivo* in rhesus macaques, using CD4-specific DARPins as a tool to provide proof of concept for their *in vivo* potential in the development of strategies against HIV.

## Materials and Methods

### Production and purification of DARPins

Expression and purification of the DARPins 57.2 and E3_5 were done as described in [8] with minor modifications to remove endotoxins. After bacterial lysis and binding of the DARPins to the Ni-NTA Sepharose (Qiagen, Valencia, CA) the column was initially washed with 30 column volumes (CV) PBS, 350 mM NaCl, 35 mM Imidazole, pH 7.4 (Wash buffer), followed by 10 CV wash buffer supplemented with 0.1% v/v Triton-X-114 (Sigma, St.Louis, MO). Subsequently the column was re-equilibrated with wash buffer, washed with 10 CV 50 mM Tris-HCl, 60% (v/v) isopropanol, pH 7.5 and re-equilibrated with wash buffer. The bound protein was eluted with wash buffer with 500 mM Imidazole and protein containing fractions were pooled and dialyzed against PBS at 4°C overnight. To lower the endotoxin content further the DARPins were rebound to Ni-NTA and the entire process was repeated. After the 2^nd^ purification the proteins were dialyzed as before. The remaining level of endotoxins in the protein preparations was quantified using the Endochrome K kit (Charles Rivers Laboratories International, Inc, L'Arbresle, France). The endotoxin concentration was 24 and 2 EU/mg protein for 57.2 and E3_5, respectively.

### Animals and treatments

Adult (5–9 years old) female Chinese rhesus macaques (*Macaca mulatta*) housed at the Tulane National Primate Research Center (TNPRC; Covington, USA) were either uninfected or chronically infected (duration of infection ≈1 year) with SHIV-RT via the vaginal route (CD4 counts: 131 - 1233, plasma viremia range: undetectable - 540000, see Table 1). Animals were injected with 30 or 300µg/kg of DARPins 57.2 and E3_5 intravenously. Blood samples (7.5 ml) and peripheral (axillary and inguinal) lymph node biopsies were collected at indicated time points after the DARPin injection. Animals were monitored continuously by veterinarians and animal care staff to ensure their welfare. The TNPRC Division of Veterinary Medicine has established procedures to minimize pain and distress. Animals were anesthetized with either ketamine (10 mg/kg) or tiletimine/zolazepam (8 mg/kg) for all procedures and given buprenorphine (0.01 mg/kg) for post-procedure analgesia. Protocols were reviewed and approved by the Institutional Animal Care and Use Committee of TNRPC (OLAW Assurance #A4499-01). Tulane National Primate Research Center is accredited by the Association for Assessment and Accreditation of Laboratory Animal Care (AAALAC #000594). Animal care and research procedures were in compliance with the regulations detailed under the Animal Welfare Act [9] and in the Guide for the Care and Use of Laboratory Animals [10].

**Table 1:** SHIV-RT infected animals that were used for the *in vivo* DARPin applications.

DARPin treatment	Animal ID*	Baseline CD4 count (cells/µl)	Baseline plasma RNA viral load (copies/ml)
**300µg/kg 57.2**	HM17	734	9,500
	HM30	781	9,000
	HM37	131	170,000
	HM24	170	130,000
	HM28	972	<30
	HM36	617	540,000
**30µg/kg 57.2**	HM20	1102	40
	HM26	190	19,000
	GT61	800	120
**300µg/kg E3_5**	HM36	802	240,000
	HM27	293	120,000
	HM28	1233	30
	HM30	982	18,000

*3 animals are listed twice, because they were treated with the two DARPins on separate occasions.

### Isolation of macaque plasma and primary cells

Plasma was isolated by centrifugation of the whole blood twice at 2000 rpm for 10 minutes at 4°C and collecting the top layer each time [11]. Plasma virus loads were then determined by the quantitative RT-PCR assay for SIV gag RNA [12]. Blood was diluted with an equal volume of cold PBS and PBMCs were isolated with density gradient centrifugation using Ficoll-Hypaque (GE Healthcare, Uppsala, Sweden). Lymph node cells were obtained by mechanical disruption and passage through 70 µm nylon cell strainers (BD Falcon, Franklin Lakes, NJ) as described previously [13].

### *In vitro* DARPin binding

For the *in vitro* binding experiments, 4×10^5^ cells were incubated in V-bottom 96-well plates (Cellstar, Carrollton, TX) with 200nM of DARPin for 20 minutes in 50 µl of FACS wash buffer (FWB) (PBS/ 1% human serum (Sigma)/ 1mM EDTA (Sigma)). Unbound DARPins were washed away by adding 150 µl of FWB, centrifuging at 2350 rpm for 2 min. This was repeated 3 times before staining.

### Flow cytometry

*In vitro* or *in vivo* binding of DARPins was measured on PBMCs, lymph node cells, and whole blood. 10^6^ PBMCs and lymph node cells were plated in V-bottom 96-well plates and 100µl of whole blood placed in a FACS tube (BD Falcon). Bound His-tagged DARPins were detected by staining for 30 minutes at 4°C (isolated cells) or room temperature (whole blood) with 1/100 dilution of anti-Penta-His Alexa Fluor 647 conjugate (Qiagen) and combined with staining for several surface markers where indicated; 1/50 dilution of FITC-anti-CD3 (clone SK7), FITC-anti-CD14 (clone M5E2), FITC-anti-CD20 (clone L27), PE- and PerCP-anti-CD4 (clone L200), PE-anti-CD123 (clone 7G3), PerCP- and APC-anti-HLA-DR (clone L243) antibodies (all from BD Biosciences, CA). “Lineage” staining was performed with anti-CD3, -CD14 and-CD20 antibodies. The combinations used for cell staining were anti-CD3/anti-CD4/anti-His, anti-CD14/anti-CD4/anti-His, anti-CD20/anti-CD4/anti-His, anti-Lineage/anti-CD123/anti-DR/anti-His and anti-Lineage/anti-CD123/anti-CD4/anti-HLA-DR. For whole blood, red blood cells were lysed with 2 ml of the FACS lysing solution (BD Biosciences) for 10 minutes. All samples were washed 3 times with FWB, fixed in 100 µl of BD Cytofix and acquired on a FACSCalibur (BD Biosciences), and analyzed using FlowJo software (Tree Star, CA). Appropriate irrelevant specificity isotype Ig negative controls were included in all experiments and typically gave MFIs of <1 log.

### *In vitro* retention of CD4 DARPins

10^6^ PBMCs from macaques that had been injected with DARPins were cultured in 48-well plates (BD Falcon) in 250 µl of R10 medium (RPMI containing 10% fetal calf serum, 2 mM L-glutamine, 10mM HEPES, 50µM β-mercaptoethanol and penicillin/strepomycin). Cells were harvested at various timepoints and labeled with anti-CD4-PE and anti-Penta-His Alexa Fluor 647 antibodies.

### *In vitro* infection assays

3×10^7^ macaque PBMCs were cultured in 15 ml of R10 medium in the presence of 5µg/ml PHA (Sigma). After 3 days, cells were resuspended at the same density in R10 medium containing 50 U/ml IL-2 (Roche, Basel, Switzerland) and plated in a 96-well flat bottom plate at a concentration of 2×10^5^ cells/well. Cells were preincubated for 1 hour at 37°C with titrated doses of DARPins, before adding 200 TCID_50_/well of SIV mac239 or SHIV-RT (SIVmac239 with the HIV RT [11], [24]). Cultures were kept at 37°C, fed with 50 U/ml of IL-2 every 2 days and supernatants collected 7 days after infection. P27 concentrations in the supernatants were quantified using the RETRO-TEK SIV p27 Antigen ELISA kit (ZeptoMetrix, Buffalo, NY), according to the manufacturer's instructions.

### Detection of free DARPin in the plasma

Opaque 96-well plates were coated with 10nM anti-human IgG (I5260, Sigma) in TBS and blocked with TBST (TBS with 0.05% tween)/1% BSA. After washing three times with TBST, 10nM PRO 542 (CD4IgG2) (Progenics Pharmaceuticals, Tarrytown, NY) was added in the blocking buffer. Following another series of washes, samples diluted in TBST were added to the wells, washed out and labeled with anti-His-tag antibody (A5588, Sigma). After a series of washes with TBST and the Tropix buffer (Applied Biosystems, Foster City, CA), CDP star (MS1000RX, Applied Biosystems) was added and the plate read on the SpectraMax Gemini EM luminometer (Molecular Devices, Sunnyvale, CA). Volumes of 100µl and incubation times of 1 hour were used for each step. All samples were run in duplicates. Pharmacokinetic analysis was performed using the NONMEM software (ICON Development Solution, Ellicott City, MD). Statistical analysis was performed using GraphPad Prism software (GraphPad Software, San Diego, CA).

## Results

### Identification of potent macaque CD4-binding DARPins

**Figure 1:**
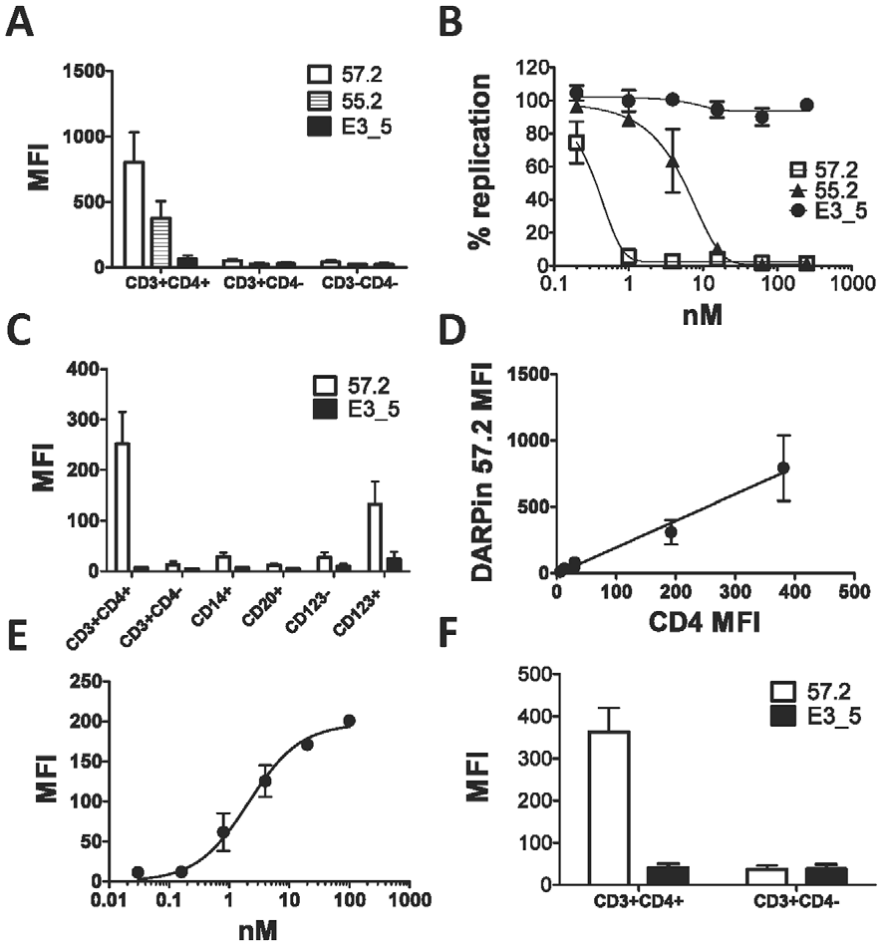
CD4-specific DARPin 57.2 binds to macaque CD4^+^ cells. **A**. PBMCs were incubated with 200nM of DARPins 57.2, 55.2 or E3_5 for 30 minutes before being labeled with antibodies for CD3, CD4 and His-tag. Mean fluorescent intensities (MFIs) of the anti-His staining on the indicated subsets identified within a total leukocyte gate from 4 independent experiments are shown (mean±SEM). **B**. PBMCs were pretreated with the indicated concentration of DARPins 57.2, 55.2 or E3_5 and then exposed to SIVmac239. 7 days later the amount of p27 in the culture supernatant was quantified by ELISA. The values shown are the means (±SEM) from 3 independent experiments and are normalized to the amount of p27 produced in the absence of inhibitors (i.e. 100% replication). **C**. PBMCs were incubated with 200nM of DARPins 57.2 or E3_5 for 30 minutes before being labeled with antibodies for CD3, CD4, CD14, CD20, CD123, HLA-DR and His-tag, as indicated. MFIs (mean±SEM) of the anti-His staining on the indicated subsets identified within a total leukocyte gate from 5 independent experiments are shown. The DC-containing fractions were identified within the Lineage^−^HLA-DR^+^CD123^−^ (CD123−, myeloid DC-containing fraction) or the Lineage^−^HLA-DR^+^CD123^+^ (CD123+, plasmacytoid DCs). **D**. The different cell types defined in panel C were stained with anti-CD4 (in the absence of DARPin 57.2) or anti-His (in the presence of DARPin 57.2) in parallel and the CD4 MFIs determined (Table 2). Each point on the graph represents a distinct cell type. Linear regression and correlation were performed using the GraphPad Prism software. An average of MFIs of the anti-His (DARPin) and anti-CD4 staining for each indicated cell type from 3 independent experiments is shown. **E**. PBMCs were incubated with various concentrations of DARPin 57.2, labeled with the anti-penta His antibody and analyzed by flow cytometry. MFIs (mean±SEM) from 2 independent experiments are shown. Curve fitting was done using the GraphPad Prism software. **F**. Lymph node cells were incubated with 200nM of the DARPin 57.2 or E3_5 and stained for His and CD4. MFIs (mean±SEM) of the anti-His staining of the gated CD4+ cell population from 5 independent experiments are shown.

We previously demonstrated the ability of human CD4-specific DARPins to cross-react with macaque CD4 [8]. This opened up a possibility of studying these inhibitors in a relevant animal model to establish whether DARPins had the potential be used *in vivo*. To move forward with the most potent DARPins, the second-series DARPins 55.2 and 57.2, which show strong binding to human CD4 and inhibition of HIV infection *in vitro* [8], were examined for macaque reactivity. Both of them, but not the control DARPin E3_5, bound CD4+ T cells isolated from macaque blood (Figure 1A). More DARPin 57.2 bound to macaque CD4+ T cells compared to DARPin 55.2. We then compared the ability of DARPins 55.2 and 57.2 to block SIV infection of primary macaque PBMCs. Both DARPins blocked SIV mac239 infection of cells derived from all three naïve animals that were tested. DARPin 57.2 had a mean IC_90_ of 0.8nM which is ∼10 times less than was required from either DARPin 55.2 (Figure 1B) or the previously studied DARPin 25.2 [8]. Therefore, DARPin 57.2 was selected for more extensive analyses and for the evaluation *in vivo* in macaques.

To further characterize the cells that were bound by the CD4-specific DARPin 57.2, we co-stained the DARPin-exposed PBMCs for various cell markers (Figure 1C). As expected, CD4^+^ T cells were stained with the highest intensity. CD123^+^ plasmacytoid DCs (Lineage^−^HLA-DR^+^CD123^+^) stained stronger than the myeloid DC-containing fraction (Lineage^−^HLA-DR^+^CD123^−^) and monocytes (CD14^+^), which were only weakly positive. B cells (CD20^+^) and CD3^+^CD4^−^ T cells were not stained above background levels. DARPin 57.2 partially blocks binding by the anti-CD4 MAb L200 used herein (Figure S1). Therefore, relative cell surface CD4 expression of different macaque leukocyte cell subsets was done in parallel on the cells from the same animal. There was a perfect positive linear association between the cell surface CD4 expression by the different cell types and staining with DARPin 57.2 (Spearman's r = 1, p = 0.003) (Figure 1D and Table 2). As expected, the amount of DARPin 57.2 binding to primary CD4^+^ cells was dependent on the input dose (Figure 1E). Binding started to plateau around 100nM and half-maximal binding was achieved at ≈3nM of DARPin 57.2. Binding fell below detection limits at concentrations below 0.3nM.

**Table 2:** Cell-surface staining for DARPin 57.2 binding (His) and CD4 by various types of macaque leukocytes.

Cell type	CD4 MFI	His MFI
**CD3^+^CD4^+^**	381±30	793±247
**CD3^+^CD4^−^**	6±1	10±1
**CD14^+^**	30±3	82±19
**CD20^+^**	13±4	36±9
**Lineage^−^HLA-DR^+^CD123^+^**	192±56	311±93
**Lineage^−^HLA-DR^+^CD123^−^**	29±8	42±3

Means±SEM from 3 independent experiments are shown.

DARPin 57.2 also bound CD4^+^ T cells in macaque lymph node cell suspensions, similar to the amount bound to blood cells (Figure 1F vs 1A and 1C). Although there was more non-specific staining with the control DARPin E3_5 in lymph node cell suspensions, due to background from the secondary anti-His antibody (data not shown), the DARPin 57.2 staining of the CD4^+^ T cells was significantly above background of the control DARPin E3_5-treated cells (p = 0.01, Mann-Whitney test). Just as in blood, staining of CD4^−^ T cells by DARPin 57.2 was not higher than the staining with a control DARPin (Figure 1F).

### DARPin 57.2 is rapidly cleared from circulation after intravenous injection

To investigate the potential of the DARPin application *in vivo*, DARPins 57.2 or E3_5 were administered via intravenous injection to clinically stable, SHIV-RT infected macaques (available from other completed studies). Overall, 6 animals received a high dose (300µg of DARPin 57.2 per kg of weight) and 3 animals received a low dose (30µg of DARPin 57.2 per kg of weight) (Table 1). The 300µg/kg injection was intended to achieve the initial plasma concentration of ≈230nM, which is similar to concentrations used in our *in vitro* experiments. As controls, 4 animals received 300µg/kg and 1 animal received 30µg/kg of DARPin E3_5. Blood samples were taken immediately before the injection, as well as 30 minutes, 2 hours, 6 hours, 24 hours, 48 hours and 7 days after the injection.

**Figure 2:**
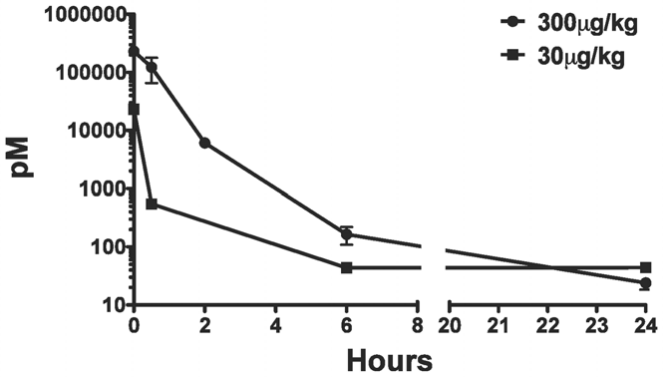
The rate of clearance of DARPin 57.2 from plasma is rapid and dose-dependent. Free DARPin 57.2 concentration in macaque plasma sampled at various times post injection was measured using a sandwich ELISA. Concentrations (mean±SEM) for both DARPin 57.2 doses are shown at each indicated time point. 6 animals received 300µg/kg and 3 animals received 30µg/kg of DARPin 57.2.

Unbound CD4-specific DARPin 57.2 was detectable in the plasma of macaques within 30 minutes after injection (Figure 2). By the 2-hour mark, only 2–4% of the injected DARPin 57.2 remained free in the plasma. By the 6-hour time point, the plasma levels of DARPin 57.2 were already below the level of detection of the assay (≈50 pM) for the low dose group of macaques. Free DARPin was still detectable in the plasma of animals that received the high dose of DARPin 57.2 at 6 hours post-injection, but fell below the detection limit by 24 hours post-injection. The log scale DARPin 57.2 concentration vs. time plot did not yield a straight line, suggesting a 2-compartment pharmacokinetic model (Figure 2). The clearance rate (CL), volume of distribution (V_d_) and α-half-life (T_1/2_) of DARPin 57.2 all increased with the increase in input DARPin dose (Table 3).

**Table 3:** Pharmacokinetic parameter estimates for DARPin 57.2.

DARPin 57.2 dose, µg/kg	β-t_1/2_, hr	α-t_1/2_, hr	V_d_, L/kg	CL, L/hr	AUC, nmoles/hr
**300**	1.30 (0.15)	0.48 (0.98)	0.16 (0.05)	0.12 (0.04)	198.0
**30**	1.34 (0.01)	0.05 (0)	0.07 (0)	0.03 (0)	8.3

NONMEM software was used to perform the analysis of the data with the IV 2-compartment model. Means (SD) for the following parameters are shown: t_1/2_, half-life (given for the two phases, as indicated); V_d_, volume of distribution, CL, clearance rate; AUC, area under the concentration-time curve. AUC was calculated using the GraphPad Prism software.

**Figure 3:**
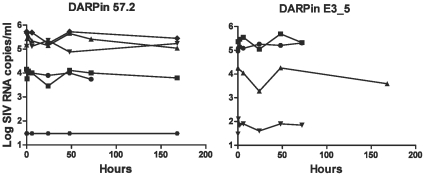
A single injection with a CD4-specific DARPin 57.2 does not lead to a change in plasma RNA viral load in rhesus macaques infected with SHIV-RT. Plasma samples were taken from the macaques at indicated timepoints after an injection with 300µg/kg of DARPin 57.2 and E3_5. SIV RNA copy numbers were determined by qRT-PCR. Each line represents an individual animal.

Since we used macaques that were previously infected with SHIV-RT, we looked at the plasma viral loads at various time points after the injection with the high dose of DARPin 57.2 to check if the one-time DARPin treatment had an effect on the infection. 3 of 6 animals had high pre-treatment virus loads (>10^5^; range 130,000–540,000 copies/ml), 2 of 6 animals had intermediate virus loads (<10^4^; range 9,000–9,500 copies/ml), and 1 animal had only 200 copies/ml (Table 1). As predicted, a single dose of DARPin 57.2 did not alter the plasma viral load compared to the DARPin E3_5 controls (Figure 3). Decreases of up to 1 log in plasma SIV RNA were observed in some animals but this was not sustained and seen in both groups of animals. To be sure that the lack of effect on SHIV-RT *in vivo* was not due to an inability of DARPin 57.2 to block SHIV-RT infection, the inhibition of SHIV-RT infection of activated PBMCs by DARPin 57.2 was measured. DARPin 57.2 also blocked the infection of macaque PBMCs with SHIV-RT (IC_90_∼10nM, Figure S2). There were also no sustained changes in numbers of circulating CD4^+^ T cells (data not shown).

### DARPin 57.2 rapidly binds cell-surface CD4 *in vivo*

**Figure 4:**
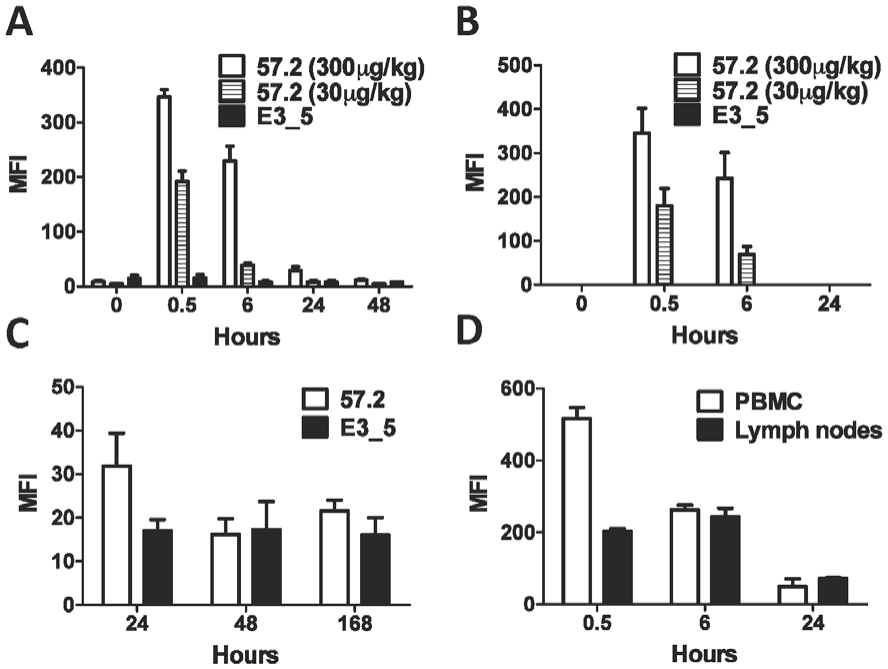
Injection of macaques with DARPin 57.2 results in transient binding to CD4^+^ cells. **A**. Blood samples were taken from macaques at indicated timepoints after an injection with DARPin. PBMCs were isolated, labeled with antibodies to CD4 and to penta-His and analyzed by flow cytometry. The MFI (mean±SEM) of His staining on CD4^+^ cells within a total leukocyte gate is shown for animals injected with 300µg/kg DARPin 57.2 (n = 6), 30µg/kg DARPin 57.2 (n = 3) or DARPin E3_5 (n = 5). Data from both doses of DARPin E3_5 was combined for simplicity. **B**. Whole blood samples taken after DARPin injection were immediately labeled with the anti-His antibody and analyzed by flow cytometry. The MFIs (mean±SEM) of His^+^ cells are shown for animals injected with 300µg/kg DARPin 57.2 (n = 6), 30µg/kg DARPin 57.2 (n = 3) or DARPin E3_5 (n = 5). Lymph nodes (**C and D**) and blood (**D**) were taken from macaques at indicated timepoints after an injection with DARPin. Isolated cells were labeled with antibodies to penta-His and CD4 and analyzed by flow cytometry. MFIs (mean±SEM) of gated CD4^+^ cells are shown for animals injected with 300µg/kg DARPin 57.2 (**C**, n = 7; **D** n = 4) or DARPin E3_5 (**C**, n = 6).

To investigate whether the circulating CD4-specific DARPin would bind CD4^+^ cells *in vivo* we sampled blood and lymph nodes at various time after injection. Initially, PBMCs were co-stained for CD4 and penta-His. Virtually all cells in the CD4^+^ gate were DARPin-positive and expressed similar levels of DARPin to that bound *in vitro* 30 minutes after DARPin 57.2 injection (Figure 4A). The level of staining was dependent on the input dose of DARPin 57.2, as seen in our *in vitro* binding studies (Figure 1E). The staining was weaker by 6 hours post-injection (but all CD4^+^ cells were DARPin positive) and was down to background levels by 24 hours for the low dose group. Weak staining was still detected at the 24 hour mark after the injection with 300µg/kg of DARPin 57.2 (Figures 4A and S3). To ensure that the DARPin binding detected in the PBMCs was not simply due to the cell-free DARPins in the plasma binding to cells during the overnight shipment of the blood to the laboratory, we carried out the His staining on whole blood samples immediately upon collection from the animals. Similar to the PBMC results, His-positive cells were detected in whole blood 30 minutes after injection with DARPin 57.2, with the level of staining decreasing by 6 hours and being undetectable after 24 hours (Figure 4B and S3). Therefore, there was little contribution from cell-free DARPins in the plasma DARPin binding to cells during the shipment.

In order to determine if the DARPins circulated from the blood to the lymph nodes, superficial lymph nodes were first sampled 24, 48 and 168 hours after the injection of DARPin 57.2 vs the control DARPin E3_5. Lymph node cells were stained for CD4 and penta-His. After 24 hours, binding by DARPin 57.2, but not by DARPin E3_5, was detected on CD4^+^ cells (Figure 4C). As seen in the peripheral blood, no DARPin binding was seen at later time points. The level of His staining (DARPin binding) was comparable to the levels seen at the 24 hour time point in the blood (compare the MFIs in panel 4A and 4C for the 300µg/kg dose). Therefore, we examined earlier time points to ascertain whether higher levels were also present in the lymph nodes earlier (as in the blood). To do this, a separate set of animals were injected with 300µg/kg DARPin 57.2 and lymph nodes and blood collected 0.5, 6, and 24 hours later. Analysis of these acute time points revealed that CD4^+^ cells in the lymph nodes were already bound by DARPin 57.2 30 minutes after the injection (Figure 4D). The levels of DARPin binding to the cells was lower in the lymph nodes than in the peripheral blood after 30 minutes, but was at similar levels after 6 and 24 hours (Figure 4D). As in blood, the entire CD4^+^ T cell population in the lymph nodes bound the DARPins.

**Figure 5:**
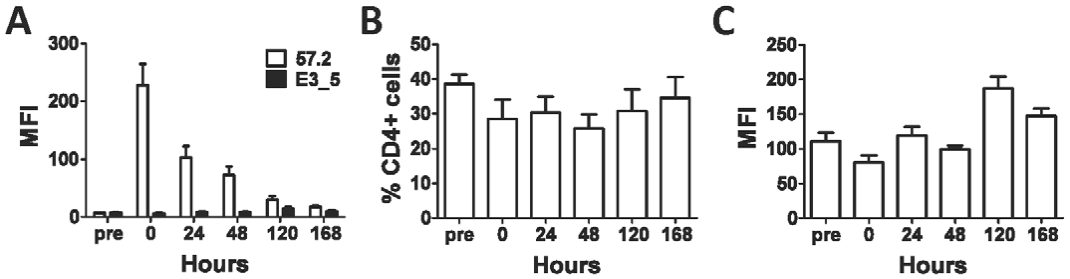
Bound DARPin 57.2 is not retained long term on macaque cells. PBMCs were collected 30 minutes after DARPin injection and cultured *in vitro* for 7 days. Cells were harvested at the indicated timepoints, labeled for His and CD4 and analyzed by flow cytometry. (**A**) The MFIs (mean±SEM) of His staining on CD4^+^ cells are shown for animals injected with 300µg/kg DARPin 57.2 (n = 4) or DARPin E3_5 (n = 3). Baseline samples were taking from the same animals immediately before the injections (pre). The percentage of CD4^+^ cells (**B**) and the MFIs of CD4 expression (**C**) for the samples described in panel **A** were also measured over time.

To better understand the mechanism by which the levels of cell bound DARPin 57.2 were decreasing with time, we isolated macaque PBMCs 30 minutes after injection with DARPin 57.2 and cultured them *in vitro*. Levels of cell-bound DARPin 57.2 decreased with time during the *in vitro* culture (Figure 5A). The cells retained DARPin 57.2 longer in culture than they did in the peripheral blood: some DARPin-bound cells were still present after 5 days in culture, while none were detected in the peripheral blood by 48 hours (Figures 4B and 5A). The decrease in DARPin binding was not due to a change in CD4^+^ cell numbers (Figure 5B). There was also no apparent decrease in cell-surface levels of CD4, although we could not accurately quantify levels of cell surface CD4 expression, because DARPin 57.2 partially blocks binding by CD4 MAbs (Figures 5C and S1).

**Figure 6:**
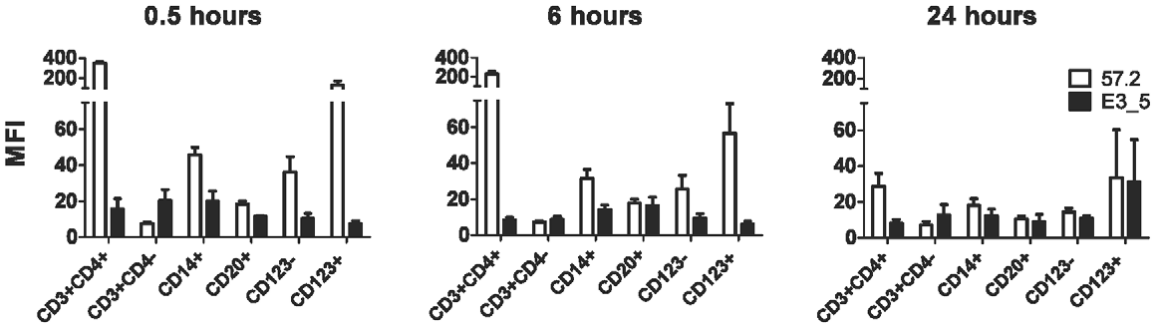
DARPin 57.2 binding to macaque leukocyte cell types *in vivo*. PBMCs were prepared from blood samples taken from macaques at 30 minutes, 6 hours or 24 hours after an injection with 300µg/kg of DARPin 57.2 (n = 6) or DARPin E3_5 (n = 4). Cells were stained the various antibody combinations (same as in Figure 1C) to define the indicated subsets. An average of MFIs (mean±SEM) of the anti-His staining for each indicated cell type is shown.

More extensive analyses were performed on the blood cells to establish which CD4^+^ cells bound the DARPins *in vivo*. Multicolor staining was performed on blood cells collected at different times post DARPin injection. *In vivo*, the pattern of DARPin 57.2 binding to cells resembled that seen *in vitro* (Figures 1B and 6). All CD4^+^ (but not CD4^−^) cells bound DARPin 57.2 proportional to the level of surface CD4 expressed. On all cell types, the greatest binding was seen at the earliest time point (30 minutes) after injection, the amounts decreasing similarly thereafter irrespective of cell type. DARPin 57.2 binding only persisted on CD4^+^ T cells after 24 hours, albeit at low levels. Therefore, DARPin 57.2 functions normally *in vivo*, binding to target CD4^+^ cells in a specific, dose-dependent manner.

## Discussion

Small proteins such as DARPins may prove to be a useful alternative to antibodies, which have disadvantages such as high cost, low stability and poor tissue penetration. Experiments done *in vitro* demonstrated that DARPins can bind their targets with high affinity and high selectivity [8], [14]–[16]. A proof of concept study was needed to investigate whether these small proteins demonstrate comparable qualities *in vivo*. We previously described the identification of human CD4-specific DARPins that specifically bound to human and macaque CD4 and inhibited HIV and SIV infection *in vitro* [8]. In this study, we characterized a more potent second series CD4-specific DARPin 57.2 for macaque reactivity and investigated its activity after a single intravenous injection into rhesus macaques.

DARPin 57.2 was chosen due to its higher levels of binding and more effective inhibition of SIV infection *in vitro*. Here we demonstrate that it rapidly binds CD4^+^ cells *in vivo*. DARPin-bound cells were detected 30 minutes after the intravenous injection not only in the peripheral blood, but also in the lymphoid tissue. DARPin could be reaching the tissue in its free form and binding resident cells there and/or we could be detecting cells that have bound DARPin in the periphery and migrated to the lymph node. If only the latter scenario were true, one would expect to find at least some tissue-resident CD4^+^ cells that are DARPin-negative, while we find that all of the CD4^+^ cells in the lymph node are bound by the DARPin 57.2. Therefore, it is likely that free DARPins can rapidly reach the lymphoid tissue and bind resident cells there.

We found that the clearance of DARPin 57.2 from the plasma was rapid, but the rate increased with the increase in dose, suggesting that DARPin elimination is a saturable process. Similarly rapid plasma clearance rate was also observed when a HER2-specific DARPin of a comparable size was used in mice [17]. The dynamics of the loss of CD4^+^ cell-bound DARPins were slower, but generally similar to that of the free DARPin in the plasma. The binding of DARPin 57.2 to primary cells was also short-lived when cells were cultured *in vitro*. Additional studies are needed to determine what happens to the CD4-specific and other DARPins after they bind their targets.

The binding levels of the DARPin 57.2 were determined by the levels of cell surface CD4 expression. Hence, binding of the DARPin 57.2 to CD4 on various cell types was likely not affected by possible cell type-dependent differences in CD4 conformations or associations of CD4 with other cell surface molecules. The vast majority of DARPin 57.2-binding cells in macaques were CD4^+^ T cells, which also had the highest levels of CD4. Unlike human monocytes, macaque monocytes have relatively low levels of CD4 expression, which resulted in low levels of binding by DARPin 57.2. In contrast, macaque CD123^+^ pDCs had higher levels of CD4 expression than monocytes or myeloid DCs [18] and consequently high levels of DARPin 57.2 binding. The functional importance of CD4 expression by these cell types and the precise role these cell types play during retroviral infection is not yet fully understood.

Monoclonal antibodies to CD4 can potently inhibit infection of T cells by primary HIV-1 isolates *in vitro*, including strains that are resistant to other types of HIV inhibitors [19]. One of the CD4-specific MAbs, Ibalizumab, substantially reduces HIV-1 RNA levels in infected people and is currently in advanced clinical trials [20]. However, single infusions with Ibalizumab did not lead to decreases in plasma viral loads at doses below 3mg/kg [21]. In our study the highest dose of DARPin 57.2 was 300µg/kg, which is slightly lower than 3mg/kg of antibody in terms of molarity. Hence, given the faster plasma clearance rate of the DARPins, a reduction in the plasma viral load was not a likely outcome. Since the animals used in the study were already infected with SHIV-RT, we checked their plasma RNA levels and confirmed our predictions that a single injection of DARPin 57.2 had no effect on the SHIV-RT plasma RNA levels. CD4-specific DARPin 57.2 did not cause a rapid flux of CD4^+^ T cells from the lymphoid tissue into peripheral blood, which was seen in Ibalizumab-treated people and macaques [21], [22]. Possibly this is caused by the cellular reaction to rapid internalization of CD4, which is induced by antibody binding but not by DARPin binding [8], [23].

Antiviral therapies targeted at CD4 can potentially have an undesired immunomodulatory effect, by either signaling through CD4 or blocking the interactions with MHC class II. CD4-specific DARPins were shown to have no effect on T cell proliferation, DC activation or overall cell viability *in vitro* [8]. Although unexpected, we were not yet able to assess DARPin 57.2 effect on immunological functions *in vivo* due to the limitations of the animal samples, but it will be looked at closer in the future studies.

Overall, our data demonstrates that DARPins can rapidly, potently and selectively bind their targets *in vivo*, even in the tissues, opening the doors for future development for novel treatment of HIV and other conditions. The high stability and low production costs could also make antiviral DARPins a promising candidate for the HIV prevention treatment, sorely needed due to the lack of effective vaccines. Topical vaginal microbicides can prevent mucosal transmission of the virus [11], [24]–[26]. Blocking the gp120 interaction with CD4 by a small molecule BMS-378806 protected most macaques from vaginal SHIV challenge, verifying that the CD4-envelope interaction is a possible target for a microbicide [25]. There are few CD4+ cells in the vaginal mucosal tissue, which makes it difficult to reliably quantify the kinetics and the effectiveness of CD4-specific DARPin binding after it is applied topically. Therefore we chose the systemic intravenous administration of the drug to provide the proof of concept that it could be used *in vivo*. Future studies in naïve animals will determine the potential of topically applied DARPins in the prevention of mucosal HIV infection.

## Supporting Information

Figure S1 PBMCs were incubated with 200nM of DARPin 57.2 or in medium alone for 30 minutes before being labeled with L200, an antibody to CD4. MFIs of the L200 staining from 4 independent experiments are shown.
(0.45 MB TIF)

Figure S2 PBMCs were pretreated with the indicated concentration of DARPin 57.2 and then exposed to SHIV-RT. 7 days later the amount of p27 in the culture supernatant was quantified by ELISA. The values shown are the means (± SEM) from 4 independent experiments and are normalized to the amount of p27 produced in the absence of inhibitors (i.e., 100% replication).
(0.47 MB TIF)

Figure S3 A. PBMCs were labeled with antibodies to CD4 and to penta-His and analyzed by flow cytometry. B. Whole blood samples taken after DARPin injection were immediately labeled with an antibody to penta-His and analyzed by flow cytometry. Blood samples were taken from macaques at 0, 0.5, 6, 24 and 48 hour timepoints after an injection with 300 µg/kg DARPin 57.2 (A and B, top rows) or 300 µg/kg DARPin E3_5 (A and B, bottom rows), as indicated. All cells within a total leukocyte gate for one representative animal are shown. This macaque, HM28, has received the two DARPins on separate occasions (Table 1).
(0.80 MB TIF)

## References

[1] Chan AC, Carter PJ (2010). Therapeutic antibodies for autoimmunity and inflammation.. Nat Rev Immunol.

[2] Weiner LM, Surana R, Wang S (2010). Monoclonal antibodies: versatile platforms for cancer immunotherapy.. Nat Rev Immunol.

[3] Stumpp MT, Amstutz P (2007). DARPins: a true alternative to antibodies.. Curr Opin Drug Discov Devel.

[4] Binz HK, Stumpp MT, Forrer P, Amstutz P, Pluckthun A (2003). Designing repeat proteins: well-expressed, soluble and stable proteins from combinatorial libraries of consensus ankyrin repeat proteins.. J Mol Biol.

[5] Binz HK, Amstutz P, Pluckthun A (2005). Engineering novel binding proteins from nonimmunoglobulin domains.. Nat Biotechnol.

[6] Sedgwick SG, Smerdon SJ (1999). The ankyrin repeat: a diversity of interactions on a common structural framework.. Trends Biochem Sci.

[7] Krzywda S, Brzozowski AM, Higashitsuji H, Fujita J, Welchman R (2004). The crystal structure of gankyrin, an oncoprotein found in complexes with cyclin-dependent kinase 4, a 19 S proteasomal ATPase regulator, and the tumor suppressors Rb and p53.. J Biol Chem.

[8] Schweizer A, Rusert P, Berlinger L, Ruprecht C, Mann A (2008). CD4-Specific Designed Ankyrin Repeat Proteins Are Novel Potent HIV Entry Inhibitors with Unique Characteristics.. PLoS Pathog.

[9] Animal Welfare Act and Regulation..

[10] Guide for the Care and Use of Laboratory Animals Committee on Care and Use of Laboratory Animals of the Institute of Laboratory Animal Resources UDoHaHS.

[11] Crostarosa F, Aravantinou M, Akpogheneta OJ, Jasny E, Shaw A (2009). A macaque model to study vaginal HSV-2/immunodeficiency virus co-infection and the impact of HSV-2 on microbicide efficacy.. PLoS ONE.

[12] Cline AN, Bess JW, Piatak M, Lifson JD (2005). Highly sensitive SIV plasma viral load assay: practical considerations, realistic performance expectations, and application to reverse engineering of vaccines for AIDS.. J Med Primatol.

[13] Teleshova N, Kenney J, Van Nest G, Marshall J, Lifson JD (2006). Local and systemic effects of intranodally injected CpG-C ISS-ODNs in macaques.. J Immunol.

[14] Milovnik P, Ferrari D, Sarkar CA, Pluckthun A (2009). Selection and characterization of DARPins specific for the neurotensin receptor 1.. Protein Eng Des Sel.

[15] Veesler D, Dreier B, Blangy S, Lichiere J, Tremblay D (2009). Crystal structure and function of a DARPin neutralizing inhibitor of lactococcal phage TP901-1: comparison of DARPin and camelid VHH binding mode.. J Biol Chem.

[16] Zahnd C, Pecorari F, Straumann N, Wyler E, Pluckthun A (2006). Selection and characterization of Her2 binding-designed ankyrin repeat proteins.. J Biol Chem.

[17] Zahnd C, Kawe M, Stumpp MT, de Pasquale C, Tamaskovic R (2010). Efficient tumor targeting with high-affinity designed ankyrin repeat proteins: effects of affinity and molecular size.. Cancer Res.

[18] Patterson S, Rae A, Hockey N, Gilmour J, Gotch F (2001). Plasmacytoid dendritic cells are highly susceptible to human immunodeficiency virus type 1 infection and release infectious virus.. J Virol.

[19] Pugach P, Kuhmann SE, Taylor J, Marozsan AJ, Snyder A (2004). The prolonged culture of human immunodeficiency virus type 1 in primary lymphocytes increases its sensitivity to neutralization by soluble CD4.. Virology.

[20] Jacobson JM, Kuritzkes DR, Godofsky E, DeJesus E, Larson JA (2009). Safety, pharmacokinetics, and antiretroviral activity of multiple doses of ibalizumab (formerly TNX-355), an anti-CD4 monoclonal antibody, in human immunodeficiency virus type 1-infected adults.. Antimicrob Agents Chemother.

[21] Kuritzkes DR, Jacobson J, Powderly WG, Godofsky E, DeJesus E (2004). Antiretroviral activity of the anti-CD4 monoclonal antibody TNX-355 in patients infected with HIV type 1.. J Infect Dis.

[22] Reimann KA, Lin W, Bixler S, Browning B, Ehrenfels BN (1997). A humanized form of a CD4-specific monoclonal antibody exhibits decreased antigenicity and prolonged plasma half-life in rhesus monkeys while retaining its unique biological and antiviral properties.. AIDS Res Hum Retroviruses.

[23] Morel P, Vincent C, Wijdenes J, Revillard JP (1993). Internalization and degradation of anti-CD4 monoclonal antibodies bound to human peripheral blood lymphocytes.. Mol Immunol.

[24] Turville SG, Aravantinou M, Miller T, Kenney J, Teitelbaum A (2008). Efficacy of Carraguard®-based microbicides in vivo despite variable in vitro activity.. PLos ONE.

[25] Veazey RS, Klasse PJ, Schader SM, Hu Q, Ketas TJ (2005). Protection of macaques from vaginal SHIV challenge by vaginally delivered inhibitors of virus-cell fusion.. Nature.

[26] Parikh UM, Dobard C, Sharma S, Cong ME, Jia H (2009). Complete protection from repeated vaginal simian-human immunodeficiency virus exposures in macaques by a topical gel containing tenofovir alone or with emtricitabine.. J Virol.

