# Supplementary figures and images for: In Vivo Binding and Retention of CD4-Specific DARPin 57.2 in Macaques
Source: PLoS One. 2010 Aug 27;5(8):e12455. doi: 10.1371/journal.pone.0012455 (PMC2929209; doi:10.1371/journal.pone.0012455)

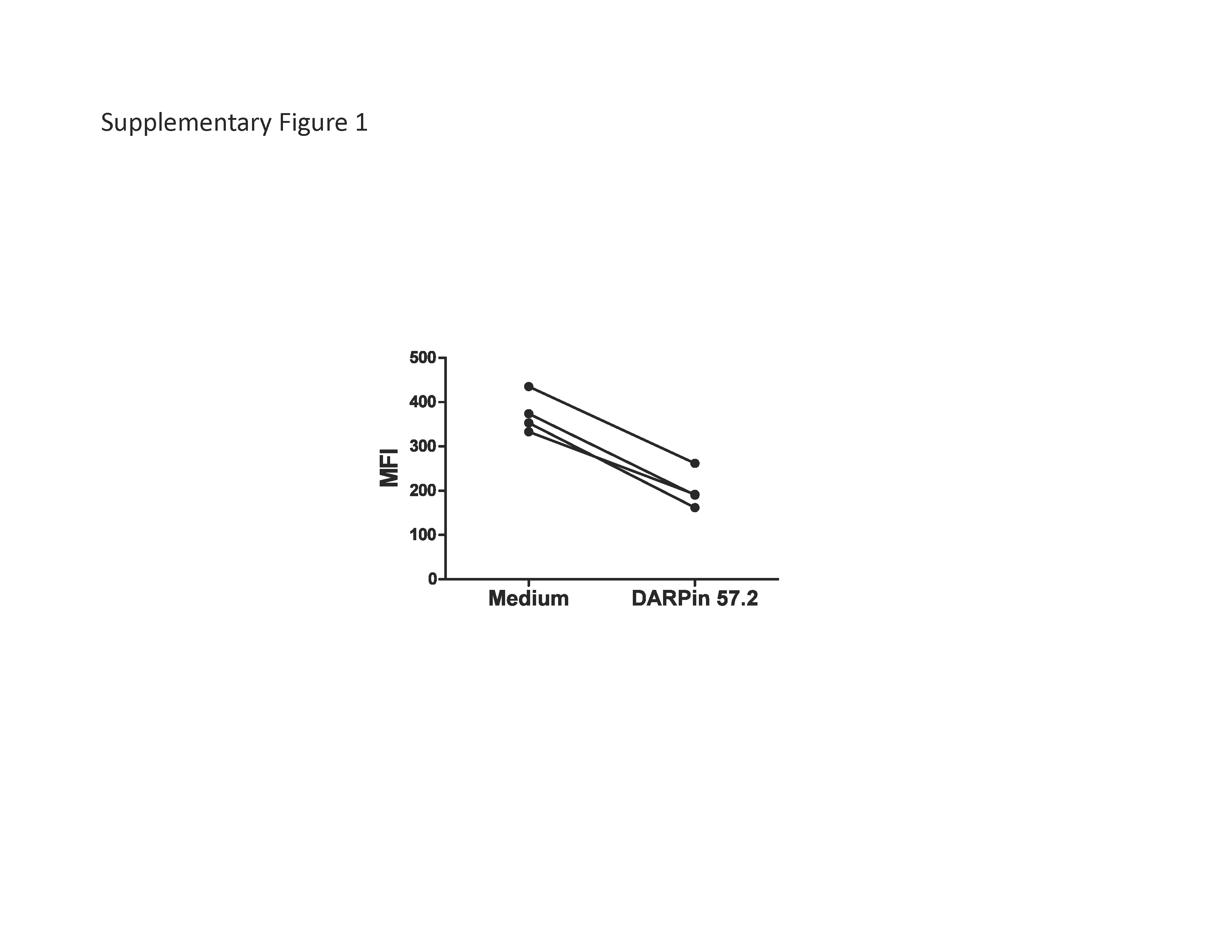

Supplement: Figure S1 — PBMCs were incubated with 200nM of DARPin 57.2 or in medium alone for 30 minutes before being labeled with L200, an antibody to CD4. MFIs of the L200 staining from 4 independent experiments are shown. (0.45 MB TIF) [file pone.0012455.s001.tif]

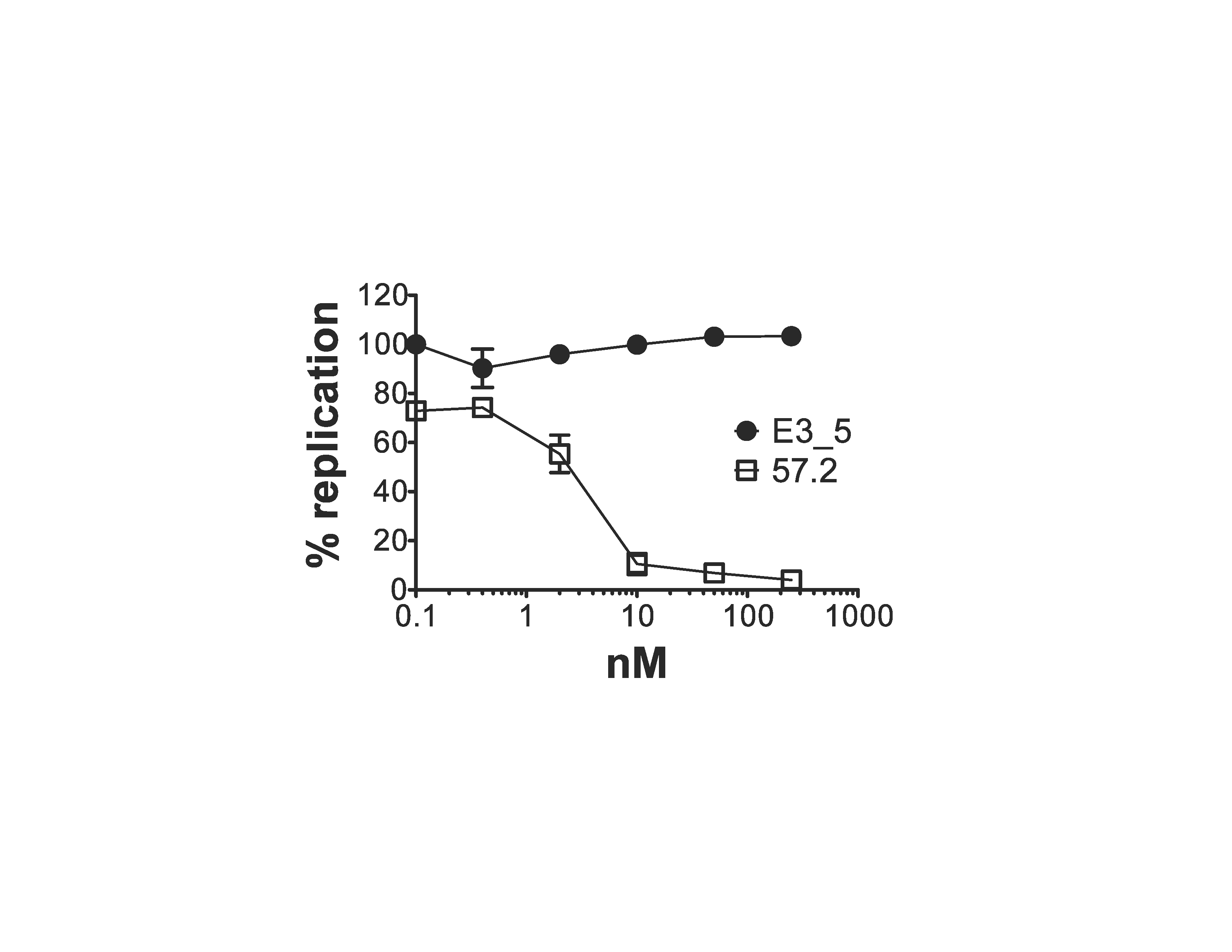

Supplement: Figure S2 — PBMCs were pretreated with the indicated concentration of DARPin 57.2 and then exposed to SHIV-RT. 7 days later the amount of p27 in the culture supernatant was quantified by ELISA. The values shown are the means (± SEM) from 4 independent experiments and are normalized to the amount of p27 produced in the absence of inhibitors (i.e., 100% replication). (0.47 MB TIF) [file pone.0012455.s002.tif]

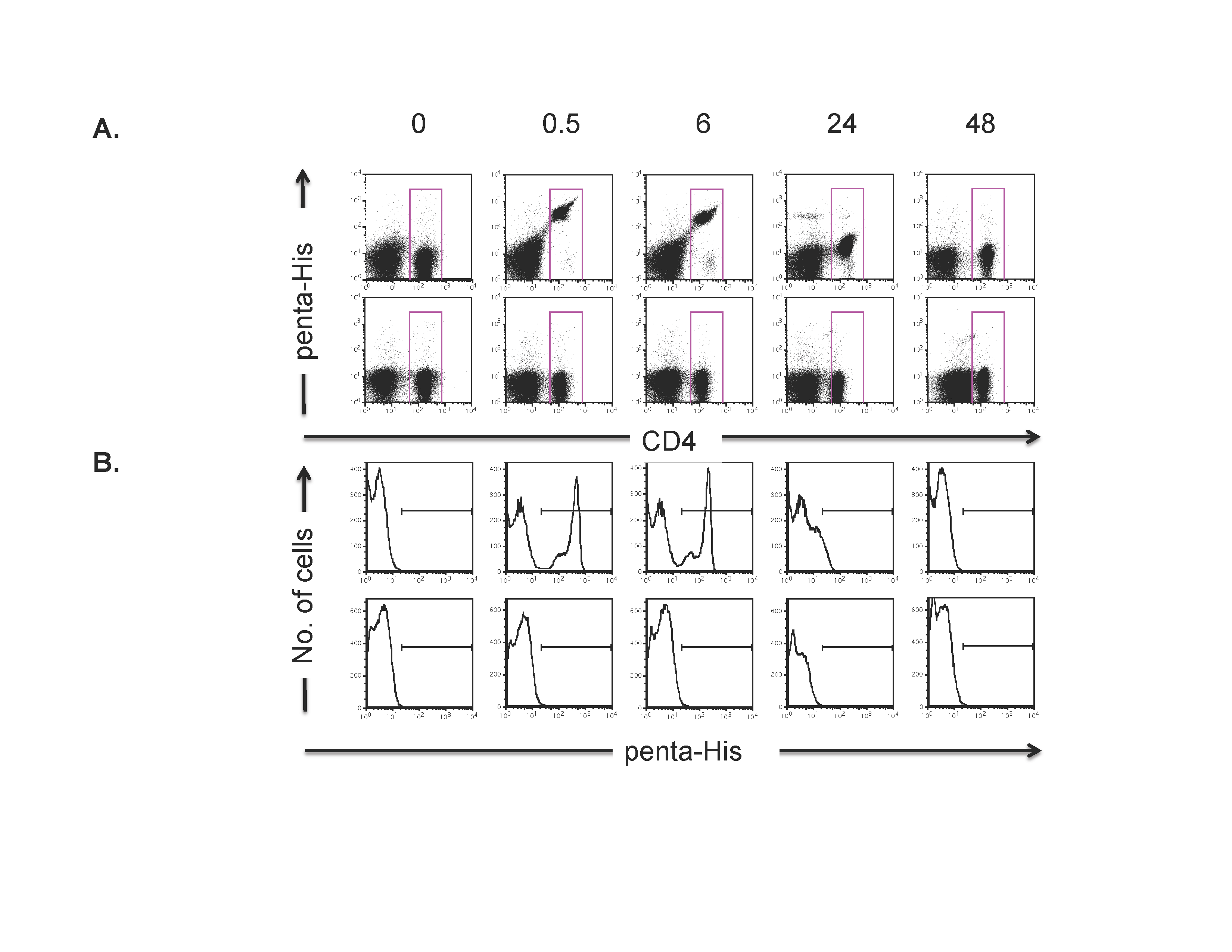

Supplement: Figure S3 — A. PBMCs were labeled with antibodies to CD4 and to penta-His and analyzed by flow cytometry. B. Whole blood samples taken after DARPin injection were immediately labeled with an antibody to penta-His and analyzed by flow cytometry. Blood samples were taken from macaques at 0, 0.5, 6, 24 and 48 hour timepoints after an injection with 300 µg/kg DARPin 57.2 (A and B, top rows) or 300 µg/kg DARPin E3_5 (A and B, bottom rows), as indicated. All cells within a total leukocyte gate for one representative animal are shown. This macaque, HM28, has received the two DARPins on separate occasions (Table 1). (0.80 MB TIF) [file pone.0012455.s003.tif]
